# 

**Published:** 1905-05

**Authors:** 


SUBSCRIPTION $1.00

PUBLISHED MONTHLY.

### ATLANTA JOURNAL-RECORD OF MEDICINE.

Successor to Atlanta Medical and Surgical Journal, Established 1855, and Southern Medical Record, Established 1870.

VOL. VII. ATLANTA, GA., MAY, 1905. No. 2.

